# Technology-Based Innovations to Foster Personalized Healthy Lifestyles and Well-Being: A Targeted Review

**DOI:** 10.2196/jmir.4863

**Published:** 2016-06-24

**Authors:** Emmanouil G Spanakis, Silvina Santana, Manolis Tsiknakis, Kostas Marias, Vangelis Sakkalis, António Teixeira, Joris H Janssen, Henri de Jong, Chariklia Tziraki

**Affiliations:** ^1^ Computational BioMedicine Laboratory (CBML) Institute of Computer Science (ICS) Foundation for Research and Technology (FORTH) Heraklion Greece; ^2^ Institute of Electronics Engineering and Telematics of Aveiro (IEETA) University of Aveiro Aveiro Portugal; ^3^ Department of Economics, Management and Industrial Engineering University of Aveiro Aveiro Portugal; ^4^ Assoc. Professor Department of Ιnformatics Engineering Technological Educational Institute of Crete Heraklion Greece; ^5^ Department of Electronics, Telecommunications & Informatics University of Aveiro Aveiro Portugal; ^6^ Sense Health Rotterdam Netherlands; ^7^ Department of Communication Stanford University San Francisco, CA United States; ^8^ ASK Community Systems GmbH Bad Schwalbach Germany; ^9^ Association of Community Elders’ Clubs (MELABEV) Jerusalem Israel

**Keywords:** mHealth, eHealth, lifestyle, health promotion, health behavior, persuasive technologies, cloud computing, personalized health monitoring, interoperability, wellness programs

## Abstract

**Background:**

New community-based arrangements and novel technologies can empower individuals to be active participants in their health maintenance, enabling people to control and self-regulate their health and wellness and make better health- and lifestyle-related decisions. Mobile sensing technology and health systems responsive to individual profiles combined with cloud computing can expand innovation for new types of interoperable services that are consumer-oriented and community-based. This could fuel a paradigm shift in the way health care can be, or should be, provided and received, while lessening the burden on exhausted health and social care systems.

**Objective:**

Our goal is to identify and discuss the main scientific and engineering challenges that need to be successfully addressed in delivering state-of-the-art, ubiquitous eHealth and mHealth services, including citizen-centered wellness management services, and reposition their role and potential within a broader context of diverse sociotechnical drivers, agents, and stakeholders.

**Methods:**

We review the state-of-the-art relevant to the development and implementation of eHealth and mHealth services in critical domains. We identify and discuss scientific, engineering, and implementation-related challenges that need to be overcome to move research, development, and the market forward.

**Results:**

Several important advances have been identified in the fields of systems for personalized health monitoring, such as smartphone platforms and intelligent ubiquitous services. Sensors embedded in smartphones and clothes are making the unobtrusive recognition of physical activity, behavior, and lifestyle possible, and thus the deployment of platforms for health assistance and citizen empowerment. Similarly, significant advances are observed in the domain of infrastructure supporting services. Still, many technical problems remain to be solved, combined with no less challenging issues related to security, privacy, trust, and organizational dynamics.

**Conclusions:**

Delivering innovative ubiquitous eHealth and mHealth services, including citizen-centered wellness and lifestyle management services, goes well beyond the development of technical solutions. For the large-scale information and communication technology-supported adoption of healthier lifestyles to take place, crucial innovations are needed in the process of making and deploying usable empowering end-user services that are trusted and user-acceptable. Such innovations require multidomain, multilevel, transdisciplinary work, grounded in theory but driven by citizens’ and health care professionals’ needs, expectations, and capabilities and matched by business ability to bring innovation to the market.

## Introduction

Non-communicable chronic diseases (NCD), primarily cardiovascular diseases, cancers, chronic respiratory diseases, and diabetes, are responsible for 63% of all deaths worldwide (36 million out of 57 million global deaths). More than 9 million of all deaths attributed to chronic diseases actually occur before the age of 60 [1]. The health and economic burden ensuing from the rising prevalence of NCDs, along with shrinking health care budgets in the face of an aging population, are major factors stifling innovation and employment opportunities [2].

The body of research is clearly directing policy to a paradigm shift, away from traditional disease treatment towards a person-centered individualized coproduction of health [3], promotion of healthier behaviors, and better coordination and management of health care [4]. Most NCDs could be better controlled and even prevented through healthier lifestyle choices made across the lifespan [5,6]. There is an urgent need for a new paradigm in health care systems [7].

Technological innovations may be key to tackling the next decade’s challenge of how to use our social and economic capital to empower and motivate individuals to engage in healthier personal lifestyle choices [8]. Information and communication technologies (ICT) can allow for a bottom-up approach. They can be used to build person-centered and community-based health services for empowering individuals with knowledge, empathic support, security, and trust [9,10] that would motivate them to choose and sustain healthier daily lifestyles [11] and well-being.

**Figure 1:**
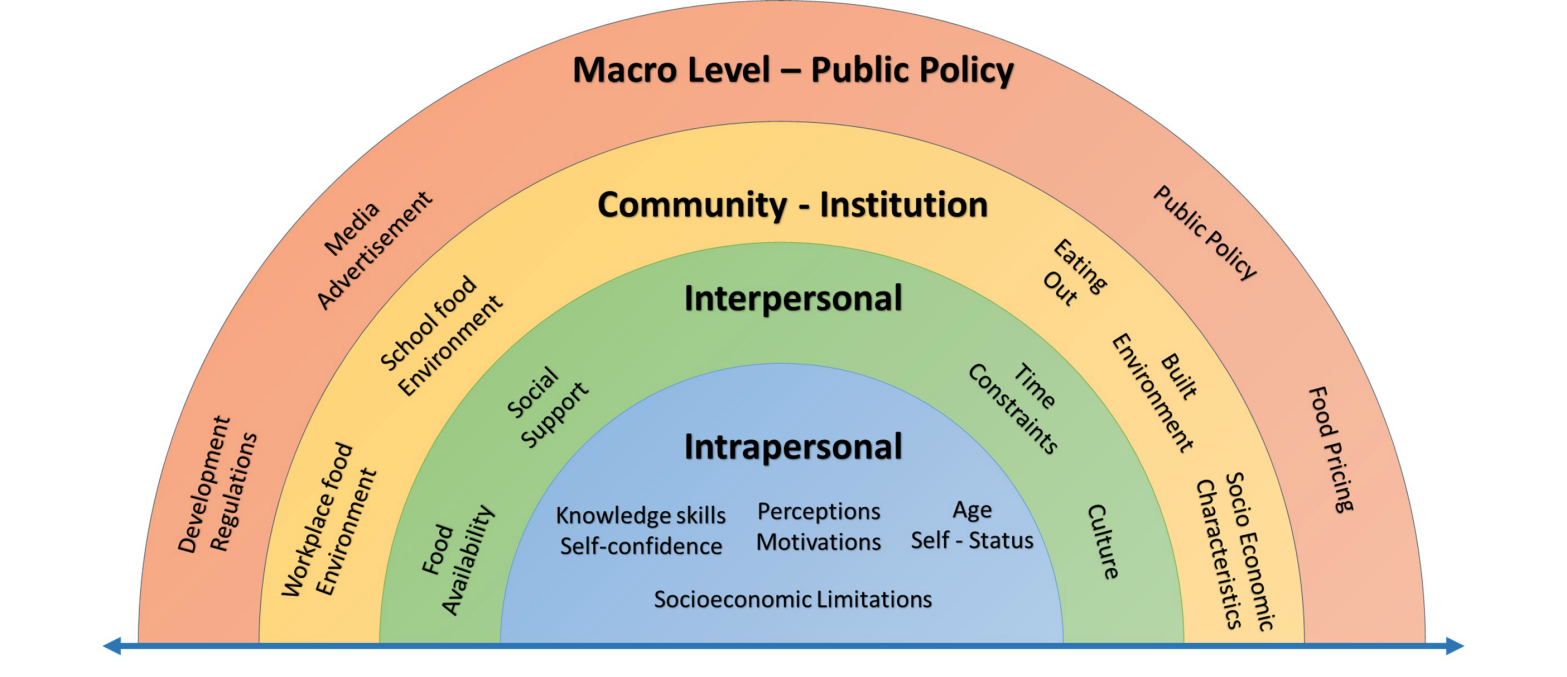
An ecological model of factors influencing life style behaviors [24].

Well-being is a widely used term encompassing various constructs [12] and addressed by several theoretical models [13] as shown in Table 1 [14-23]. The ecological model (see Figure 1) toward healthier lifestyles, well-being, and wellness takes into account multiple predisposing factors, requirements, and barriers such as intrapersonal variables [24] (eg, personality, health beliefs, knowledge, attitudes, and skills), interpersonal processes and their likely interactions with genetics, as well as community and macro/public policy levels factors. The arrow in Figure 1 extending across the four levels suggests that factors or barriers extend into and interact across various levels.

**Table 1:** Dimensions and determinants of wellness according to models in the literature [13].

Physical	Emotional physiological	Social	Intellectual	Spiritual	Occupational	Environmental	Reference
X	X	X	X	X	X		[14]
X	X	X	X	X	X	X	[15]
X	X	X	X	X	X		[16]
X		X	X				[17]
X	X	X	X	X	X	X	[18]
X	X	X		X	X	X	[18]
X	X	X	X	X			[19]
X	X	X	X	X	X		[20]
X	X	X	X	X		X	[21]
X	X					X	[22]
X	X	X				X	[23]

Research has shown that interventions for change are likely to take place, not in the traditional health care system, but rather in what sociologists have labeled “enabling spaces” [25]. Virtual or spatial enabling spaces provide the opportunity for “mingling, observing, and lingering” where peer learning and teaching is actualized within a community. The goal of this work is to identify and discuss key innovations that may foster healthy lifestyle choices and well-being [26-31].

## Methods

We follow a bottom-up approach for the review of state-of-the-art and beyond state-of-the-art theory and practice on personalized technology and innovations for empowering individuals to self-perceived well-being and healthy lifestyles. The question we are trying to answer is the following: What are the main sociotechnical challenges that need to be successfully addressed in delivering state-of-the-art, ubiquitous eHealth and mHealth services in the 21^st^century, including citizen-centered wellness management services for the large scale ICT-supported adoption of healthier lifestyles?

Our goal is to generate evidence on community-based, citizen-centered interventions and review key enabling technologies that can support research and bridge the gap between ICT health and social care systems [32]. We do so by critically reviewing selected state-of-the-art work in the field in order to identify some of the areas in need of further research. We present key concepts and themes that would contribute to consolidating the body of knowledge and propel further developments on the multidisciplinary sociotechnical aspects of health-related data collection, modeling, representation, and unification in a complex, person-centered ecosystem. We emphasize these innovations that are, in our opinion, necessary in order to sustain personalized healthy lifestyles and well-being for the public.

In more detail, this paper is structured as follows. First, we present the state-of-the-art technologies in the areas of personalized health monitoring systems, activity profiling, lifestyle capturing, and infrastructure supporting services, highlighting challenges in development and deployment of such technological solutions. Second, from the stakeholders’ standpoint, we present a vision on specific key concepts that would channel a leap forward, that is, person-centered ICT-based innovations, ushering in a new generation of services enabling changes at the personal level and a paradigmatic shift in health and social care systems. These technologies could be used to achieve personalized healthy lifestyles, wellness, and well-being through individual empowerment and engagement and effective self-management. We highlight and discuss specific innovations that would be able to support personalized healthy lifestyles and well-being for the public. Third, we discuss important drawbacks and pitfalls that must be addressed for the effective use of high-quality technological solutions that are able to serve people through secure, safe, and trustable innovative services to take place.

## Results

### State-of-the-Art Approaches, Techniques, and Technologies

Personalized health systems and pervasive mobile monitoring can enable sensing, mining, and learning of human behaviors and intentions. Examples include personalized mobile information delivery, context aware social networking, device and environment customization, serious games and entertainment, education, safety, and mobile business [33]. Their potential in the domain of lifestyle changes towards wellness, well-being, and self-management of chronic diseases, at the individual, organizational, and community levels is enormous, as can be seen from the following examples.

### Personalized Health Monitoring Systems

#### mHealth and Smartphone Platforms

Smartphones are rapidly becoming the central computing, sensing (large number of sensors embedded), and communication platform for deploying personalized wellness mobile apps and services [34-36]. Smartphone capabilities include two types of sensing technologies: hardware-based sensors (physically present in the device) and software-based sensors (virtual sensors fusing multiple hardware sensors’ data). These include accelerometers, global positioning system (GPS), digital barometer, microphone, camera, ambient light sensor, digital compass (Magnetometer), assisted GPS, proximity sensor, near field communication, global navigation satellite system, finger print reader, and many more [37].

Existing research [37-40] has evaluated smartphones as a method for delivering key components of established and empirically validated behavioral weight loss treatment, with an emphasis on adherence to self-monitoring. Results show that smartphones can be advantageous for optimizing adherence to self-monitoring and to the inclusion of behavioral strategies for evidence-based interventions. They have also been used to track social interaction, sleep, and physical activities, and to provide intelligent feedback promoting better health and well-being [41]. Currently, there are also several systems allowing developers to build apps and services on top of a smartphone’s sensing capabilities [42-44].

#### Intelligent, Ubiquitous, and Smart Applications

Sensor-enabled mobile phones have the potential to collect *in situ* continuous sensor data that can dramatically change the way health and wellness are assessed and monitored, as well as how self-management of health conditions is made and care and treatment are delivered. New classes of applications are being explored both in academic- and industry-based research centers.

Advances in human-centric sensing are being fueled by the combination of sensor data and classification models to recognize human activities [45] and environmental context [46]. These apps correlate sensing information with personal health data and encourage users to be physically active and meet their related goals. Research is being conducted on using unobtrusive monitoring technology to study how mood changes are correlated with both social interactions and non-sedentary work style [47]. Examples exist on studying smoking habits and cessation, but the existing systems have low levels of adherence to key guidelines [48]. We found that only a few apps provide recommendations to the user for proven treatments such as pharmacotherapy, counseling, and/or a quit line.

A major challenge is to develop innovative personalized technologies that can help individuals maintain healthy lifestyles and wellness by keeping track of their everyday activities. Today, we have a plethora of apps and services supported by modern smartphones and mobile apps using embedded or external sensing devices. Most of these smart well-being tracking systems focus on capturing physical activity, ﬁtness, and sleep patterns [43-48]. Major smartphone manufacturers include in their app suite dedicated proprietary software to allow people to self-monitor their health consistently, by logging and checking exercise, activity, sleep, food intake, and heart rate, among others [49-51]. All these apps are able to connect with specialized clock-like body-worn devices [52-54], allowing users to constantly record their activity and receive live feedback in real-time mode even if they do not carry their smartphone with them.

A recent study analyzed the content of many popular free apps related to physical activity and compared them against existing guidelines and fitness principles already established by the American College of Sports Medicine (ACSM) [55]. Results show that very few are evidence-based and respect the guidelines for aerobic activity, strength/resistance training, and flexibility, set forth by ACSM. The authors clearly identified a gap in safely and effectively using evidence-based apps to start a physical routine program, develop fitness, and lose weight. Nearly all the apps, although technically well designed, did not meet the basic recommendations of ACSM for exercise prescription, and therefore, would not be suitable for beginning exercisers. Thus, users are advised to select apps with extreme caution. Use of mobile technologies may have the potential to transform care delivery across populations and within individuals over time. However, such devices and/or services may need to be tailored to meet the specific patient and doctor needs [56,57].

### Personalized Activity Profiling and Lifestyle Capturing

#### Human Activity and Lifestyle Recognition

Human activity recognition is a challenging problem for context-aware systems and apps. Research in this field has mainly adopted techniques based on supervised learning algorithms, but these systems suffer from scalability issues with respect to number of processed activities and richness of contextual data [58]. Efforts have been made toward the development of a unified framework for activity-recognition behavior-based analysis, and action prediction for the daily routine of people. A novel approach for enhanced classification of activity recognition data includes a game-like app used to reward physical activity and encourage healthy lifestyle choices [59,60]. Increasingly, context-aware systems are focusing on multiple domains besides physical activity, such as mental and social well-being [61].

Recent research has shown that emotions can now be recognized more accurately by machine learning algorithms than by laypersons [62]. Consequently, approaches for the automated recognition of different aspects of our social lives, like empathy, dominance, and non-verbal behavior have progressed rapidly [63-65]. Bringing these novel technologies into health apps will likely increase their effectiveness by further tracking important mental health parameters and personalizing services based on them.

#### Personalized Health Assistance and Interaction Support Platforms

In recent years, there have been many developments in the area of natural user interfaces, from touch screens to assistants integrating dialogue capabilities. Intelligent assistants that interact with users via conversational natural spoken language can provide them with meaningful and easily understandable information and advices (eg, about their prescribed medications) [65-68]. User-oriented, intuitive interaction is necessary in order to overcome the barriers of app acceptance. Special attention should be given to specific groups of people, like chronic patients and the elderly. Cardiac is such a prototype for a conversational assistant for chronic heart failure patients by using natural spoken dialogue [69]. Its helps patients managing their treatment and monitoring their health by using natural spoken dialogue over the telephone or with in-home systems to conduct regular checkups to collect relevant information on their condition. Similar research has focused on developing appropriate technical solutions, such as fission, information output arrangement, and organization modules for multimodal systems [70,71].

### Infrastructure Supporting Services

#### Cloud for eHealth

Large amounts of data currently sit in different silos within health and social care systems. If these data were to be released in an appropriate manner and used effectively, they could transform the way care is provided [71]. Bringing together and correlating data among different and heterogeneous data sources would allow inference of new knowledge [72,73]. Cloud computing is emerging as a model for enabling convenient, on-demand network access to a shared pool of configurable resources that can be rapidly provisioned and released with minimal management effort or service provider interaction [74]. Institutions and medical professionals who frequently do not have enough storage and computing resources can manage their biomedical information through apps built on these type of services, accessing advanced computing infrastructures that they could not afford otherwise [75-78]. While many companies, like Google, IBM, Amazon, and Microsoft were early adopters of cloud computing, its application to biomedicine has only recently been proposed, mainly for bioinformatics applications.

Nevertheless, cloud computing is also benefiting the health care sector and the wellness management domain [77]. It can provide effective, on-demand, high elasticity access services to citizens from anywhere at any time. Data sharing on the cloud is quickly becoming vital for organizations and social users alike. In a survey by InformationWeek [79], nearly all organizations shared their data in one way or another with 74% sharing data with customers and 64% sharing data with suppliers.

The benefits that motivate organizations to move towards this direction include higher productivity and better time management. Health care providers are willing to store and share electronic medical records via the cloud and hence remove the geographical dependence between health care provider and patient [80]. Sarathy and Muralidhar [81] reviewed the impact of the Internet on data sharing across many different organizations such as government agencies and businesses. Butler [81] describes the issues of data sharing on the Internet where sharing information can allow users to infer details about users. Feldman and Patel et al [82] discuss the important benefit of data sharing in terms of public health, in particular for education and professional development.

However, the cloud is susceptible to privacy and security attacks, many of which occur from within the cloud providers themselves as they have direct access to stored data [83]. Malicious insiders represent one of the major issues affecting the cloud [84,85]. There is considerable work on protecting data from privacy and security attacks. The National Institute of Standards and Technology has developed guidelines to help consumers protect their data on the cloud [86]. Encrypting data before storage is an effective way to prevent unauthorized users from accessing sensitive data [87]. However, plain encryption techniques are not enough, especially when considering the scenario of sharing data among a large group of users [88].

#### Standards for Health and Interoperability

With the adoption of electronic health services, an opportunity exists for the creation of longitudinal health records that span many decades and aggregate data from multiple health care organizations’ source for delivering care. The need for standards for the representation and exchange of medical information becomes apparent. As deﬁned by the Health Information Management Systems Society, an electronic health record (EHR) “is a longitudinal electronic record of patient health information generated by one or more encounters in any care delivery setting.” According to the report of the National Institutes of Health (National Center for Research Resources) on EHRs, three main organizations create standards related to EHRs: the Health Level Seven (HL7), the Comité Européen de Normalisation - Technical Committee (CEN TC) 215, and the American Society for Testing and Materials (ASTM). HL7, which operates in the United States, develops the most widely used health care‒related electronic data exchange standards in North America. CEN TC 215, which operates in 19 European member states, is the preeminent organization developing health care information technology (IT) standards in Europe. Both HL7 and CEN collaborate with the ASTM, which is mainly used by commercial laboratory vendors. EHRs today use both technical and clinical standards. However, EHR vendors have implemented only some standards, while having a great deal of variation in their implementations, which results in systems that cannot interoperate and for which secondary use of data, for example, research and epidemiology, is difﬁcult. Current EHR systems, due to their evolution over time, are often just an electronic representation of the previously used paper records. They are highly idiosyncratic, vendor-speciﬁc realizations of patient record subsets. They adopt few, if any, health information standards and very rarely accommodate controlled terminologies where they might be sensible.

A variety of health care communication standards has also been developed during the last decade. Their goal is to improve the interoperability and the connectivity among devices, computerized systems, tablets, smartphones, and health information systems, standardizing the content and structure of the information exchanged. Within the EU region, qualified medical devices and software depend on whether or not the device or software falls within the scope of the Medical Devices Directive (ie, 2007/47/EC). Currently, the directive states that software can indeed be qualified as a medical device, but unfortunately it does not specify what exact kind of software will meet the medical device definition per se.

The Continua Health Alliance guidelines [89] describe a set of standards to allow vendors, solution developers, and sharers of various types of health-related information to easily share their data. Some of the communication protocols supported by these guidelines are Bluetooth, ZigBee, USB, Wi-Fi, and Li-Fi. There are also tools available allowing vendors, programmers, and engineers to build on this approach. These resources are important in order to realize the communication interoperability recommended by the Continua Guidelines. Such examples are the Wipro Continua Toolkit (which enables medical devices to become compliant with the Continua specified protocol, ie IEEE 11073-XXXXX, and contains a Wipro Continua Agent and a Wipro Continua Manager), Stollman BlueHDP (health device profile)+USB dongle [90], Toshiba Bluetooth HDP stack and application programming interface, and the Advanced and Adaptive Network Technology (ANT+) [91].

#### Service-Oriented Architecture and Service-Oriented Device Architectures

People do not experience technology. They experience services, made available by technology [92], or that should be the case. However, due to the heterogeneity in hardware and communication interfaces, interoperability is still a major concern in today’s distributed architectures. This concern is particularly addressed by Service-Oriented Architecture (SOA) [93,94]. SOA implementations focus on principles such as separation of business logic from the underlying technology, efficient use and reuse of resources, compliance to standards, granularity and modularity, delivery, and monitoring of services. Service-Oriented Device Architecture (SODA) resulting from an adaptation of SOA intends to provide some level of abstraction to physical devices, simplifying external access [95-97].

#### Semantic Web/Linked Data and Ontological Approaches

The Semantic Web aims at a machine interpretable view of the World Wide Web. It focuses on structuring the Web based on content categorization to improve aspects such as automatic discovery, composition, invocation, and interoperation of services. The Semantic Web stack includes a number of technologies and standards aimed at this purpose (eg, Resource Description Framework or Web Ontology Language (OWL), SPARQL).

In the domain of health and wellness, a state-of-the-art example using semantic description is the GoPubMed [98] portal—a knowledge search engine for biomedical texts. At its core, GoPubMed uses two ontologies, GeneOntology [99] and MeSH [100] Ontology, but also allows users to create a custom ontology for specific searches. Another state-of-the-art example is NextBio, an ontology-based semantic framework based on gene, tissue, and disease ontological representation [101]. The use of ontology-based information extraction is explored in systems such as MedInX to allow semantic search of medical information originally buried in unstructured written text [102]. This approach is applicable both to private and public data, such as the one available on the Web.

One key problem with working with ontologies is that application ontologies tend to jeopardize the aim of ontology-based semantic interoperability to work with generalized references in the given domain. With respect to the biomedical arena, a suite of quality-checked, interoperable domain ontologies is being developed [103]. Yet, we need easy-to-use techniques to create application ontologies from the semantic information stored within those domain ontologies. This is a transformation of the bench-to-bed problem, since we have to bridge the gap between highly theoretical ontological representations to functionality-oriented ontologies.

### Innovations to Support Healthy Life Styles and Well-Being

In this section, we discuss person-centered developments for the adoption of healthier lifestyles that are beyond the state-of-the-art. We emphasize the perspective of overarching ICT-supported innovations to influence the way health is maintained. Acquire, profile, represent, persuade, assess, manage, connect, and respond are some of the essential capabilities/functions/services needed to create innovative user-centered approaches.

A change toward healthier lifestyles is both attainable and urgent, given the current situation in most western world economies. Research shows that ICT resources and health behavior interventions can be brought together to make it feasible and effective, improve health outcomes, and bring down costs [104]. Behavior aware computing and pervasive technologies include novel patterns and activities relevant to individuals’ health and well-being. Still, the problem of identifying behaviors from an individual’s own annotated data, collected by multiple sources (eg, smartphones, sensors, and smart clothes) remains [105]. Care and most health-related practices continue to be caregiver-centered, despite recent efforts [106].

In order to go beyond the state-of-the-art, the user must be brought in as an active participant to care processes and related decision-making processes. To allow informed decision making, we need to advance intelligent user profiling and understandable instruction information to users. We need to enable interaction with Internet-of-Things (IoT) resources and services to retrieve and transmit information to users in simple everyday devices, such as mobile phones, smart watches, and home appliances [107]. This implies advancement in technologies and systems able to continuously track health-related data, despite coverage and communication means.

Healthy lifestyle change is attainable but not always easy to achieve, and sustaining behaviors, including use of ICT-based health-related programs, might be particularly challenging. Crucial evolution is needed in the process of making the essential information available and usable for the users, helping a person make a decision, and developing and deploying usable empowering end-user services that are accepted and enjoyed [108].

#### Acquiring User’s Activity and Behavior In Digital Form

Many techniques have been proposed to automatically recognize human activities. Most important approaches use either statistical or symbolic reasoning. However, to date, these methods have mainly been considered separately. Proposed statistical activity recognition techniques differ on the kind and number of used sensors, activities considered, learning algorithms adopted, and many other parameters. One research direction focuses on using video with sound, image, and scene recognition software [109]. Other activity recognition techniques are based on data acquired from body-worn sensors (eg, motion tracking and inertial sensors, cardio frequency meters) and on the application of statistical learning methods. Early attempts in this field were mainly based on the use of data acquired from multiple body-worn accelerometers [110].

One of the main limitations of these early systems was the fact that they did not consider contextual information (eg, current location, environmental conditions, and surrounding objects) that could be used to derive user activity. On the other hand, the recognition of complex activities like social ones (eg, work meetings, friendly chat) is particularly challenging and is hard to achieve by the use of solely statistical methods. Indeed, complex activities can be better recognized by considering constraints and relationships among context data that can be neither directly acquired from sensors nor derived through statistical reasoning alone.

As a result of these constraints, there is a growing interest in the use of ontology-based techniques to automatically recognize complex context data such as human activities. While most activity recognition systems rely on data-driven approaches, the use of knowledge-driven techniques is gaining increasing interest. Research in this field has mainly focused on the use of ontologies to specify the semantics of activities and ontological reasoning to recognize them based on context information. In particular, in the area of pervasive computing, OWL DL has been used to build activity ontologies and to recognize activities based on context data [111,112]. The ontological approach to activity modeling consists of a knowledge engineering task to define the formal semantics of human activities by means of the operators of the ontological language. Ontological reasoning is used to recognize that a user is performing a certain activity starting from some facts (eg, sensor data, location of persons and objects, properties of actors involved). Previous research has led to the definition of an OWL DL ontology for the activity recognition domain called COSAR [113], which is published on the PalSPOT [114] project website. This particular ontology definition was used to refine the predictions of statistical activity recognition systems by means of symbolic reasoning. The experimental evaluation of the effectiveness of this ontology-based activity recognition service, using a dataset collected in a smart-home setting, revealed the importance of including temporal reasoning in ontological techniques to effectively recognize activities.

#### Intelligent User Profiling for Healthy Lifestyles and Wellness

Intelligent user profiling [115] enables the collection of information from different sources to construct individual profiling models, with the objective of optimizing information delivery from health professionals to individuals in a personalized empowering environment. The most common user profile contents are the following: short/long-term user interests; knowledge, background, and skills; goals and user objectives or purpose with respect to the application; behavior; interaction preferences; and individual characteristics [116-119]. One such model is the OCEAN model [119-121].

User context is of great importance in the area of health, healthy lifestyles, and wellness management to characterize a situation of an entity [122]. Profiling information representation is following keyword-based models where interests are represented by weighted vectors or keywords [123]. Weights usually represent the relevance of the word for the user or within the topic. Another way to represent user interests is through topic hierarchies. Goals or intentions can be represented in different ways, either based on multicriteria analysis techniques [115] or Bayesian networks [124,125]. In this representation, nodes represent user tasks and arcs represent probabilistic dependencies between tasks. Given evidence of a task performed by the user, the system can infer the next most probable task and hence the user’s goal.

To obtain and build a user profile, the information can be provided explicitly by the user or be obtained through implicit observation of user actions. The simplest way of obtaining information about users is through the data they submit via forms or other user interfaces provided for this purpose. Especially for patients, their profile information is commonly assessed by patient-reported outcome measures (PROMs) including health-related quality of life information. PROMs can be defined as “reports coming directly from patients about how they function or feel in relation to a health condition and its therapy, without interpretation of the patients’ responses by physician or anyone else” [126]. These instruments embrace a broad range of health dimensions such as physical, psychological, and social functioning [127]. On the other hand, in order to implicitly collect information about user’s actions, their actions should be logged and patterns should be discovered using data mining, information retrieval, or machine learning techniques [128-131]. However, to be able to discover patterns, the user behavior should be repetitive, and the behavior observed should be different for different users. When no information about a user is available, a stereotype can be used as the default information, enabling the classification of users as belonging to one or more of a set of subgroups, and also the integration of the typical characteristics of these subgroups into the individual user profile [132].

#### Persuasive Technology

Adopting healthy lifestyles is one of the biggest opportunities for preventing chronic diseases. A great interest among researchers is how to explore the use of ICT to change behaviors, a field that has been baptized persuasive technology [133,134]. However, in spite of a wealth of psychological theories on health behavior change, modeling them and embedding them into effective ICT solutions has proved to be difficulty, in great part due to their qualitative nature. Moreover, those theories offer population-based models that do not take into account individual differences. In light of this, there is great promise in combining tracking technologies with statistical techniques to learn an individual’s susceptibility to specific persuasive strategies.

Early findings suggest that combining tracking and statistical learning, user’s psycho-emotional characteristics and context information, could personalize persuasive technology [135], greatly improving its effectiveness [134]. For instance, where some users might be susceptible to facts and statistics, others will be more easily convinced by emotions and personal stories. By having context aware systems simultaneously, the right moment of messaging can also be selected (eg, when the received message is most necessary and actionable). The field is open for the exploration and exploitation of right-mood right-time right-place feedback that could be more effective than real-time feedback. The goal is to provide evidence of the adherence of people to healthier behaviors using ICT-enabled persuasive services to support safe, secure, seamless monitoring and persuasive guidance, and personalized assistance from lifestyle improvement to primary, secondary, and tertiary prevention and care [136].

#### Biomarkers in Cognitive Function Assessment for Wellness

Lifestyle choices greatly affect healthy aging. Still, accurate quantitative diagnostic tests assessing neurodegenerative diseases and cognitive function remain a challenging problem [137]. Currently, diagnosis is mostly conducted by eliminating other possible causes and is usually performed through a combination of psychological tests. However, there has been a significant effort towards the manufacture and market of low-cost consumer brain-computer interface devices [138,139] capable of capturing brain signals and decoding conscious thoughts or emotions, such as movement intentions, facial expressions, excitement, engagement, and frustration. The underlying technology of these wireless devices is based on the ability of the electroencephalogram (EEG) to capture oscillatory brain activity reflecting mean electrical patterns characterizing different brain processes, in terms of cognitive engagement [140-144]. Capturing such activity may be attributed to biomarkers since they reflect the integrity of cognitive pathways. Hence, such information has great potential for use in disease prognosis, in progression monitoring, and even in self-improvement/self-regulation, based on functional connectivity analysis algorithms [143] and neurofeedback approaches [145], respectively. Areas of successful applications include neurodegenerative diseases such as dementia [144], mental disorders such as schizophrenia [146], neurodevelopmental disorders such as autism [147], and even addictive disorders such as alcoholism [148]. Although there is still a long way to go in order to clinically validate these approaches, such technologies are ushering in an era where we will be able to take advantage of the brain’s wiring.

#### Disease Management Using Smart Environment and Personalized Mobile Monitoring Services

New care models incorporating advanced ICTs have the potential to provide service platforms able to improve health care, personalization, inclusion, and empowerment of the individual [149]. Continuous management of diabetic patients can help in achieving effective glucose control and lifestyle changes leading to improved nutrition and healthy levels of physical activity, and to recognize and treat complications [150]. The goal is to empower patients by increasing their ability to self-manage, improve the quality of their life and the overall management of their condition but also to reduce the risk of developing complications and decrease utilization of health care resources [151]. However, there is lack of robust evaluation, including controlled trials on the effectiveness of these powerful new ICT tools [152].

In an Ambient Intelligence environment, various wireless and wired sensor technologies can be integrated, allowing the user to control and interact with the environment. Such innovative systems [152-154] are able to augment surrounding spaces with smart features to allow better lifestyle monitoring and wellness for a user, regardless of geographic location. If the user is inside a hospitalized environment, the system is able to alter the interfaces to support personalized interaction for the medical personnel. A major challenge related to caring chronic patients is the early detection of exacerbations of the disease. To address this challenge, recent research [155] presents a real-time remote monitoring framework enhanced with semantic technologies (within an Ambient Intelligent environment). It provides personalized, accurate, and fully automated emergency alerting systems that smoothly interact with the personal health professional, regardless of their physical location in order to ensure in-time intervention in case of an emergency.

#### Smart and Medical Grade Networking for eHealth

Future health informatics for personalized eHealth services rely on innovative technologies for transparent and continuous collection of evidence-based medical information at any time, from anywhere and despite the coverage and availability of communication means. In light of this transformation and change, ICT serves as the catalyst for accelerating the preparedness of all traditional players and to assist all actuators to evolve in envisaged future health care models and systems. Disruption and delay tolerant networking is a novel approach for next-generation eHealth information services where end-to-end homogeneous networking connectivity is not always available [155,156]. For future eHealth and mHealth services, the goal is to provide in-transit persistent information transfer and/or storage allowing uninterruptible services overcoming network instabilities, incompatibilities, or even absence for long periods of time [2].

#### Personal Health Record Platforms for Digital Patients

Personal health record (PHR) systems are an important, innovative, and constantly evolving area that empowers patients to take a more active role in their own health and make informed decisions. One of the most promising aspects of PHRs is to improve health care delivery and reduce costs. The primary goal of a PHR system is to provide the patient with the ability to maintain and manage their PHR, a “systematic collection of information about an individual’s health and health care, stored in electronic format” [129-131,157]. PHRs provide a complete summary of patients’ health history, enhance accurate clinical diagnosis, and empower patients to manage their own health [131]. The interconnection of data sources is an important aspect for modern PHRs. The collection of heterogeneous health parameters, for accessing, sharing, and analyzing long-term multilevel health data, including clinical, genetic, sensor, human behavior, and activity, enables clinical analysis, prediction, and prevention for the individual citizen. Triggering intervention on detection of conditions that may lead to health deterioration for preventive care becomes possible. Recently, there have also been development efforts that aim to implement a useful, effective, and intelligent PHR framework that will satisfy the variety of health environment needs and foster an optimal user experience [107,157].

### Drawbacks and Pitfalls

Technological innovations to foster personalized healthy living and wellness could dramatically improve our ability to sense, monitor, and manage our health status and contribute to a change of paradigm in health care. Yet, misuse of technology and, above all, personal data generated through them, may lead to substantial deleterious effects. Below, we discuss important drawbacks and pitfalls that must be addressed for the use of services supported in such technologies to be perceived as secure, safe, and trustable and to be adopted at a greater level than today.

#### Security and Trust

The objective of a secure system is to protect sensitive health information from unauthorized access, manipulation, and misuse. The protection goals that ascribe the requirements of a secure system are defined by the following objectives: authentication, authorization, confidentiality, integrity, accountability, and non-repudiation. It is essential that intelligent pervasive health care solutions are developed and correctly integrated to assist health care professionals in delivering high levels of patient care [158]. It is equally important that these solutions are used to empower patients and relatives for self-care or wellness management and to provide seamless, trustable access to health care services. One of the major challenges to be addressed is how to ensure security and privacy. Adapt-lite [159] and Hide-n-sense [97] are examples of how security mechanisms can be applied in mHealth services.

The face of health care is changing as new technologies are being incorporated into the existing infrastructure. The population of new technologies could possible dramatically improve our ability to detect, monitor, and address lifestyles and wellness but also could cause substantial deleterious effects. Adoption of technological innovation to support personalized healthy lifestyles and well-being depends on the extent that public concerns about privacy, confidentiality, and security of online data are addressed. Today there is a rapidly growing market of online apps and social media tools for health, with little focus on the issues of ownership and protection of data [160].

A huge volume of data is generated and is expected to increase by the use of future services either intentionally by users or automatically by networked devices, such as information appliances and sensors. Product developers and policy makers will need to proactively balance public concerns about privacy protection with the information-sharing needs of some business models and public health programs. Policies, regulatory and otherwise, will need to keep pace with technologic innovation.

A cloud infrastructure, for example, is susceptible to many privacy and security attacks [161]. As a result, many hospitals, biomedical research groups and health care organizations are reluctant to adopt this technology as a privacy breach with respect to the patient information managed under their jurisdiction could be devastating, especially in terms of cost [162]. This is highlighted in the work of Seong Han et al [163], who report that the biggest obstacle hindering the progress and the wide adoption of the cloud are privacy and security issues associated with it. This is further evidenced from a survey carried out by IDC Enterprise Panel [164], where most users pointed out security as the top challenge. Nevertheless, significant research still needs to be done to ensure that only selected and trusted resources are used, for example, a trust-based security framework as proposed by van’t Noordende et al [165].

In order to foster trust, users must receive clear and objective information about the benefits and risks associated with the use of the new systems and services. Since sensitive health and lifestyle data are going to be processed, measures must be taken in order to protect the data, stored and in transit, against unauthorized disclosure or access, accidental or unlawful destruction, or accidental loss or alteration according to Article 17 (1) of the Data Protection Directive (Directive 95/46/EC). When one considers the requirements imposed by standards or other regulations, such as the requirements for the fair and lawful processing of data established by the European Data Protection Directive [166] or the requirements of HIPAA (Health Insurance Portability and Accountability Act) [167] in the United States, it becomes evident that it is crucial for health-related data to be kept confidential from anyone unless authorized by the patient or some emergency regulations. Moreover, establishing a legal framework of future services is required in order to ensure lawful and fair data processing for personal medical and clinical data. On January 25, 2012, the EU Commission published its draft General Data Protection Regulation 2012/0011(COD). The Regulation’s stated intention is to build a stronger and more coherent data protection framework in the EU that will resolve current legal uncertainties, put individuals in control of their own data, and bring greater legal and practical certainty for organizations that are subject to the legislation. The EU Commission has indicated that it aims to have in place a revised legislative framework by 2016.

#### Quality and Effectiveness

Many of these innovations will be complex and would need to be networked to and operate in conjunction with other existing services and applications. Ensuring interoperability is fundamental in order to minimize the risk of disruption when integrating heterogeneous services. This can be achieved by using quality and evaluation processes throughout the development life cycle and stable architectural description plans [168]. In order to foster effectiveness, we must redesign the methods and context for performing real-life trials to ensure the evaluation of these technologies and stretch the limits of existing methods of creating and applying robust interventions. The ultimate goal is to identify and answer the health and social impact of the intervention at the population level. The challenge will be to develop consensus methods and metrics around this fundamental question. Today, many services widely deployed have been validated using methodologies inherited from traditional health interventions and many times do not have empirical evidence of benefit [169].

## Discussion

### Principle Considerations

Advances in the theory of the brain, neuroscience and the mechanisms of behavior modification [137,143] and the power of positive psychology offer an opportunity to health care providers and managers, as well as citizens for real empowerment to change the biggest and yet modifiable factors that contribute to the top chronic diseases. The economic impact of these chronic diseases and the “ongoing economic uncertainty brings into sharp focus the fact that current health care models are financially unsustainable” [2]. Moreover, the traditional health care system alone has not been able to accomplish the shift toward healthier lifestyles and the expected reduction in chronic diseases and their associated economic burden.

ICT offers a promising new approach toward wellness throughout the lifespan, by fostering and supporting the active and meaningful participation of individuals in their health management. At the same time, it can maximize the information flow with clinicians and open new horizons for individualized medicine approaches based on multilevel personal data.

In order to realize this paradigm shift towards citizen empowerment and engagement in health and well-being production, it is clear that a new generation of ubiquitous and convergent network and service infrastructures is required. Their function will be to sustain the construction and the deployment of highly personalized, scalable, flexible, manageable, context-aware, dependable, and secure services incorporating resources in a holistic seamless ecosystem [150]. These infrastructures need to support an Internet of things, dynamically combining devices, communication and delivery systems and services. Virtualization of resources remains an important research driver, enabling the delivery of services independently from the underlying platform [2,170]. The focus is on the use of pervasive mobile technologies so that scientists and researchers can easily design, share, and execute simulations.

Enormous challenges remain and multidisciplinary approaches are required to engineer an information infrastructure that ensures privacy and supports intelligent and personalized user-computer interaction, innovative conversional agents and assistants with emphasis on a citizen’s engagement and empowerment. Computer-delivered interventions can lead to better behavioral health outcomes, post-intervention improvements in health-related knowledge, attitudes, and intentions as well as positive changes in health behaviors such as dietary intake, tobacco use, substance use, safer sexual behavior, binge/purging behaviors, and general health maintenance [171].

The primary challenge for relevant future innovations is to utilize and modify existing technology with an emphasis on mobile communication technology and devices to (1) create new models of impact on chronic disease development able to empower individuals to adopt and sustain changes towards healthy choices (eg, nutritional habits, physical activity, stress management, sleep, anxiety, depression, meaningful social interaction, and networking) and to inform decisions makers and funding bodies on the best available choices; (2) create positive and personally motivating marketing strategies for individuals to become engaged and motivated to improve their own health on the following pillars: sensing and assessing individualized health behaviors and status, providing feedback tailored to psychosocial profiles, incorporating end users in the development of the technology, creating strong relationships for ownership and empowerment, and connecting the individual to community-based support/capacity structures to boost, encourage, sustain, and integrate their own self-regulating efforts; (3) use and improve available health supporting services for sensing and early detection of risky health behaviors; (4) develop an ecosystem of community-based capacity to support individuals who choose to be coproducers of healthy lifestyles in all levels of health care delivery (primary, secondary, tertiary); (5) provide a “proof of concept” of this model of integrated person-centered healthier lifestyle and well-being through ICT; and (6) create appropriate business models of community-integrated elements for health coproduction across cultural milieu, able to foster the scaling up of results and to bring social and technological innovation to the market.

In achieving such a paradigmatic shift, it is critical to provide smart ubiquitous services and systems advancing the current state-of-the-art in a number of domains relevant for risk factors that may influence directly or indirectly an individual’s wellness and well-being and lifestyle behaviors: (1) smart and ubiquitous sensor fusion and Internet of things services to allow health-related information to be aggregated and transmitted for remote monitoring and response, to support personalized/individualized multilevel patient/citizen-centered health care services; (2) assistant/coaching services for wellness management enabling citizens to manage their health and well-being; (3) intelligent decision-making support services through the combination of understanding with the ability to influence decisions by communicating subjective values, helping to select interventions, assessing outcomes, and providing feedback to the user; (3) novel mechanisms and services to support self-efficacy confidence that the individual can perform a given behavior, including decision making and a belief in their ability to change the situation; (4) ubiquitous just-in time support services with mechanisms that incorporate known principles of health behavior change, based on the user’s biopsychosocial profiling with representations of motivation cues and techniques for overcoming barriers to change; (5) ecosystems and personalized health promoting services managing multilevel information and medical data from multidisciplinary domains, including the health and social care systems; (6) service infrastructures able to support unified data access, management, presentation, sharing, and security, based on specific user requirements and engagement; (7) linkage services, able to connect government agencies and authorities to allow official public health structures to use the coming innovation for preventing diseases and promoting health through organized efforts and informed societal choices of public and private organizations, communities, and individuals; (8) train for the health and social care workforce in the utility of ICT; and (9) programs able to generate large population-based evidence on the efficacy and cost effectiveness of this paradigm shift.

### Conclusion

This paper focuses on innovative and meaningful ways to empower and engage individual citizens into sharing knowledge and awareness and becoming coproducers of their health and wellness through the adoption of eHealth and mHealth technology. This personal choice to being coproducer of one’s own health and wellness will have a ripple effect in both the way health is produced and maintained as well as on the kind of models and enterprises that can sprout in the communities of individual users.

Despite the innovative approach and expected results, such a paradigmatic shift cannot happen by overriding existing wellness and chronic disease management practices and services. Change needs to happen with and within current arrangements. In order to support and strengthen such movement, a number of trends would need to be supported by future research: (1) Evidence depends on the implementation of large-scale solutions based on innovative services, by means of a patient-centered approach that must also address the issue of comorbidities and their management; (2) Outcomes must be validated, strengthening the evidence for chronic disease management, through robust health technology assessment for effectiveness, quality, and continuity of care, personalization, cost-efficiency, satisfaction of the users, and transferability; (3) The rollout of services should be preceded by the development of guidelines to identify profiles of patients who may benefit from the provision of such services (condition, age, severity of the condition, comorbidity, socioeconomic status, and any other relevant factors); (4) Conditions for building on top of existing solutions and reuse, when possible, established and scientifically validated methodologies must be studied, and public national and regional authorities that act today as eHealth service providers must be involved; (5) Scale-up will depend on the dissemination and exploitation of good practices and coaching of early adopters and followers on how to effectively connect to the existing traditional health care system; (6) Assuring security and privacy in digital environments through stronger and transparent mechanisms, as predecessors of safety and trust, is of fundamental importance; providers need to realize that trust is a factor perceived by the user based on a dynamic process and it must be cared over time; and (7) The complex, changing needs of a mobile society will increasingly push for an agenda where all the above aspects will meet under cross-border deployment of health and wellness management services, and issues of interoperability, quality, and care standards will regain importance.

The challenge today is to facilitate the adoption and use of ICT tools that go beyond the current state-of-the-art, as described in this paper, not only by the engaged young population but also by the unengaged older people who are in urgent need of behavior change towards healthier life habits. We need health data to flow back to its owners so they can manage their own health and improve their own health metrics. The evidence so far suggests that these services need to be easily accessible, enjoyable, connected to people’s everyday life activities, and adapted to the end-user’s physiological and age-related changes. It is also important to educate the medical and social systems on the use and benefits of these technologies for achieving the desired health outcomes. By introducing such innovations within the health care system, we can promote community capacity and social engagement through better and healthier behavior and lifestyle of individuals.

## Abbreviations

ACSM: American College of Sports Medicine
ASTM: American Society for Testing and Materials
CEN-TC: Comité Européen de Normalisation - Technical Committee
EHR: electronic health record
GPS: global positioning system
HDP: health device profile
HL7: Health Level Seven
ICT: information and communication technology
IT: information technology
NCD: non-communicable chronic diseases
SOA: service-oriented architecture
SODA: service-oriented device architecture
